# Ammonia Distribution Measurement on a Hot Gas Test Bench Applying Tomographical Optical Methods †

**DOI:** 10.3390/s19040896

**Published:** 2019-02-21

**Authors:** Bernhard Fischbacher, Bernhard Lechner, Bernhard Brandstätter

**Affiliations:** Kompetenzzentrum—Das Virtuelle Fahrzeug Forschungsgesellschaft mbH, Inffeldgasse 21A, 8010 Graz, Austria; bernhard.lechner@v2c2.at (B.L.); bernhard.brandstaetter@v2c2.at (B.B.)

**Keywords:** ammonia, deep ultraviolet, selective catalytic reduction, spectroscopy, tomography

## Abstract

Measuring the distribution of gas concentration is a very common problem in a variety of technological fields. Depending on the detectability of the gas, as well as the technological progress of the sector, different methods are used. In this paper, we present a device and methods to detect the ammonia concentration distribution in the exhaust system of diesel engines in order to increase the performance of the exhaust aftertreatment system. The device has been designed for usage on a hot gas test bench simulating exhaust gas conditions. It consists of multiple optical beams measuring ammonia line concentrations by applying nondispersive absorption spectroscopy in the deep ultraviolet region. The detectors consist of photodiodes allowing high sampling rates up to 3 kHz while providing a high signal-to-noise ratio. A detection limit of only 1 ppm has been achieved despite the short path length of only eight centimeters. The obtained line concentrations form an inverse problem. The methodology of the tomographic techniques is described in detail in order to best solve the inverse problem and obtain the ammonia concentration distribution images for each time step.

## 1. Introduction

### 1.1. Legislation

NO_X_ emission reduction is still an important issue in diesel engine development. The introduction of the current EURO 6d temp and upcoming emission legislation including the new real driving emission (RDE) procedure forces manufacturers to reduce drastically NO_X_ emission [1]. The previous regulations, using the New European Driving Cycle (NEDC), led to an optimization of emission in the cycle, but did not result in the expected emission reduction in real driving. Especially for NO_X_ emissions, the difference between real driving and the cycle is particularly large [2]. The reasons therefore are on the one hand a very mild NEDC cycle with low vehicle speed and low engine load conditions and a purposeful optimization of the emission behavior on the type-approval test cycle. Engine operating conditions creating high NO_X_ emissions are typically high engine loads with high combustion temperatures and air excess, which are not well represented in the NEDC cycle [3].

### 1.2. Selective Catalytic Reduction

The most promising exhaust aftertreatment technology in order to reduce NO_X_ emissions is selective catalytic reduction (SCR). SCR technology uses a urea-water solution (UWS) injected into the exhaust gas. The UWS is converted to ammonia (NH_3_) through thermolysis and hydrolysis and acts as a reduction agent for NO_X_ in the SCR reaction [4,5]. The reaction between NH_3_ and NO_X_ takes place in the SCR catalyst converter, eliminating up to 99 percent of the NO_X_ emissions [6]. In order to prevent ammonia tailpipe emissions caused by unintended overdosing of the UWS, the SCR catalyst is followed by an ammonia slip catalyst. Ammonia itself is a toxic emission gas to be prevented as best as possible. The current legislation does not fully cover the limitation of ammonia emissions. Yet, NH_3_ emission is only regulated for heavy duty trucks and buses with diesel or gas engines. The tailpipe emissions are defined with a maximum gas concentration of 10 ppm. The overall limitation of ammonia emission for all vehicle classes and engine types seems advisable [7,8,9].

**Figure 1:**
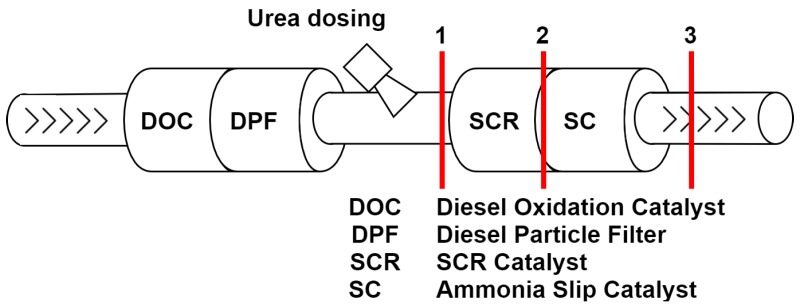
An exemplary arrangement of the exhaust aftertreatment system components of a diesel engine with SCR technology.

The ammonia concentration distribution at the catalyst converter is a key parameter for best conversion performance [10,11,12]. Only a homogeneously- and uniformly-distributed ammonia concentration ensures the effective usage of the whole catalyst cross-section. Local under- or over-dosing of ammonia leads to an insufficient NO_X_ conversion rate or ammonia slip. Figure 1 shows an example of a possible exhaust aftertreatment system for diesel engines using SCR. The red lines indicate the cross-sections of interest for investigations on the ammonia concentration distribution. These positions represent the distribution entering the SCR catalyst (1), leaving the SCR catalyst (2), and the tailpipe gas distribution (3). Commonly-used methods to determine the ammonia distribution extract gas from various discrete positions in the cross-section [6]. This method has poor temporal resolution, taking several hours to determine the concentration distribution of the cross-section for one operating point. Further, the ambient conditions of the whole experiment including the engine and all other components of the exhaust system are presumed to stay constant for that period of time. In addition, extractive methods have to deal with measurement uncertainties due to possible chemical reactions within the analysis path. In order to obtain moderate geometrical resolution, the traversing of the cross-section needs a high number of closely-located measurement points. Moreover, the gas extraction has to be sampled isokinetically to cover a defined region of the cross-section.

This new in situ approach enables sampling rates up to 3 kHz, which is faster by a factor of 10^7^ compared to extractive methods and enables the investigation of dynamic processes in the exhaust aftertreatment system to increase the system performance. In situ optical measurement techniques are not intrusive and therefore offer the great advantage of not manipulating the original experiment. In addition, the measurement quantity is acquired directly at the point of interest.

### 1.3. Ammonia Concentration

For ammonia detection, various measurement techniques are available [13,14]. Within absorption spectroscopy, the light absorption by the gas of interest is wavelength specific. By detecting the light absorption at the sensitive wavelengths with possibly no other gas species absorbing in this spectral range, the line concentration can be determined. The concentration estimation follows the Beer–Lambert absorption law (Equation (1)). The absorbance *A* is defined as the logarithmic ratio of the incident light and the detected light and is used to calculate the line concentration *c* using the calibrated absorption coefficient α and the path length *l* (Equation (1)). The developed measurement system applies nondispersive techniques using an optical bandpass filter in the desired spectral range in combination with a photodiode.

(1) $$A=\ln\left(\frac{I_{0}}{I}\right)=\alpha \cdot c\cdot l$$

### 1.4. Tomography

The line concentration per optical path represents the integral of the gas concentration along the path. By applying as many optical paths as possible in different positions and angles through the cross-section, the information gain can be increased only limited by the optical access to the cross-section. In this application, 20 light paths are instrumented through the cross-section. The resulting inverse problem is reconstructed by applying least squares regression and Tikhonov regularization [15,16], leading to 2D images of the ammonia concentration distribution in the measurement cross-section.

## 2. Materials and Methods

### 2.1. Absorption Spectroscopy

Ammonia absorbs light mainly in three spectral ranges: in the DUV, near-infrared, and mid-infrared regions (Table 1). The measurement requirements—sampling rate greater 100 Hz, detection limit of about 1 ppm ammonia—identified the deep ultraviolet (DUV) spectral region to be the best choice. The developed measurement system must be capable of detecting the ammonia concentration before, as well as after the SCR catalyst converter. As shown in Table 1, the absorption coefficient defining the system sensitivity is highest in the DUV region, being 10-times higher than in the MIR and 10,000-times higher than in the NIR region. The cross sensitivity to nitric oxides and aromatic compounds is no issue for measurements on the hot gas test bench, but must be considered for migration of the system to the engine test bench.

For the application on the hot gas test bench—dealing only with ammonia and no other gases with cross sensitivities in the spectral absorption region of ammonia—the nondispersive measurement principle has clear advantages. Due to the broad spectral region of interest, the light power detected is increased, leading to higher signal amplitudes and hence better detection limits and a higher signal-to-noise ratio (SNR). In addition, the higher signal amplitude enables faster sampling rates due to the lower integration time of the detector. This combination enables sampling rates of up to 3 kHz and allows very detailed time-resolved investigation of the flow properties in the pipe.

### 2.2. Nondispersive Single-Beam Detection System

**Figure 2:**
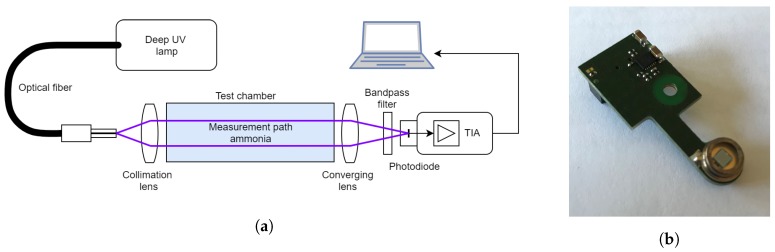
Nondispersive absorption spectroscopy measurement setup; (**a**) measurement setup of a single optical beam; (**b**) picture of the detector board containing the optimized transimpedance amplifier and the photodiode.

**Figure 3:**
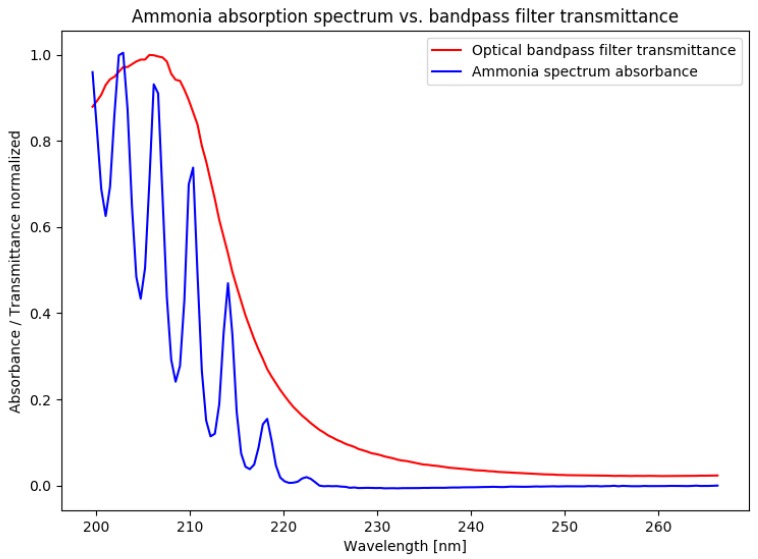
Nondispersive detection principle showing the normalized absorption spectrum of ammonia and the normalized transmittance spectrum of the optical bandpass filter.

A single measurement path (Figure 2a) of the system consists of a deuterium gas discharge lamp as the light source for several optical beams connected via a multifurcated solarization-resistant UV fiber-optic cable. The optical access to the measurement cross-section is realized via a fused silica lens collimating the emerging light from the fiber. On the detector side, a fused silica lens is used as a converging lens focusing the light on the detector. The detector consists of an optical bandpass filter and a photodiode directly connected to an optimized transimpedance amplifier (TIA) (Figure 2b). The bandpass filter has a central wavelength of 205 nm with an FWHM of 10 nm. Ammonia absorbs in the region of 200–225 nm; hence, the filter forms an integral over the spectral range of interest and realizes the nondispersive measurement principle (Figure 3). The properties of the UV-enhanced photodiode are considered when designing the TIA with respect to amplification, speed, and stability.

### 2.3. Beam Arrangement Design

**Figure 4:**
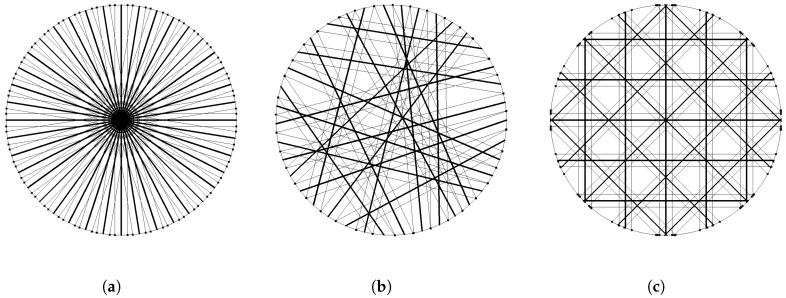
Multi-path measurement system: In order to gain maximum information out of the 20 optical beams, the geometrical arrangement is varied. (**a**) shows the worst case as the baseline for reconstruction error. (**b**) shows the result of an optimization algorithm in order to reduce the reconstruction error. (**c**) shows the chosen arrangement that is mechanically feasible.

The result of a single optical beam through the cross-section is a line concentration in physical terms and a projection in mathematical terms. The number of beams through the cross-section is limited by geometrical constraints enabling optical access to the pipe. Twenty beams are defined to be a reasonable number as a trade-off between optical access and information gain for the reconstruction algorithm. Since the arrangement of beams through the cross-section directly influences the information gain, different scenarios are investigated. In order to benchmark arrangement candidates, the base line is defined by a worst case scenario (Figure 4a). This arrangement on the one hand has the best sensitivity in the center, but lacks information about the distribution in outer regions. The best arrangement fulfilling the mechanical constraints (Figure 4c) is a regular beam arrangement with four angles and five beams per angle. The information gain of the regular arrangement has to be tested against a possibly best beam arrangement. Therefore, a variety of concepts for optimal beam arrangement, as well as optimization problems are taken into account. Different attempts are executed to find an analytical solution of the information content by only investigating the system matrix representing the geometrical properties of the beam arrangement without the need for representative test data and statistical analysis. Known methods [16,17] analyzing the so-called resolution matrix formed from the system matrix and its regularized pseudo-inverse or analysis of the distribution within the sinogram space representation are implemented and used as the cost function within the optimization problem. However, for the system under test, the optimization results do not correlate with simulation results testing the candidates with suitable test data. The reconstruction error using test data from CFD simulation being similar to the expected concentration distributions show very little correlation to the optimization problem. The mathematically-optimized arrangement (Figure 4b) results from minimizing the reconstruction error using simulation test data, as well as random distributions of Gaussian circular concentration peaks. Comparing the reconstruction error using test data for all three arrangements in Figure 4, the worst case (Figure 4a) is about 15 percent worse than the optimized solution (Figure 4b), while the regular arrangement (Figure 4c) is only about 3 percent worse than the optimized solution (Figure 4b). Overall, the regular beam arrangement (Figure 4c) is a well-performing candidate in terms of information gain.

### 2.4. Tomographical Reconstruction

The inverse problem of limited data hard field tomography can be solved by applying least squares regression and Tikhonov regularization (Equation (2)). The setup can be described as a linear equation system where the geometry of the beam arrangement is summarized in the system matrix A. The low number of projections compared to the number of image pixels of the cross-section led to a severely under-determined equation system. This fact demands modification and extension of the equation system in order to increase reconstruction quality. One possibility is to insert a priori information into the equation system. For the application of gas distribution measurement, good prior information is smoothness and non-negativity. Using Tikhonov regularization, the system can be augmented by additional equations to span the null space [15]. In general, the formulation of Tikhonov (Equation (2)) consists of the measurement term ${∥A\cdot x-b∥}^{2}$ and the regularization term ${∥\lambda \cdot L\cdot x∥}^{2}$. The measurement term is represented by the system matrix A and the measured projection vector b. The regularization term consists of a weighting factor λ and the regularization matrix L, here a discrete gradient operator to introduce smoothness as prior information.

(2) $$x_{\mathrm{reconst}}=\underset{x\geq 0}{\arg\;\min}\left\{{∥A\cdot x-b∥}^{2}+{∥\lambda \cdot L\cdot x∥}^{2}\right\}$$

### 2.5. Weighting Parameter Optimization

**Figure 5:**
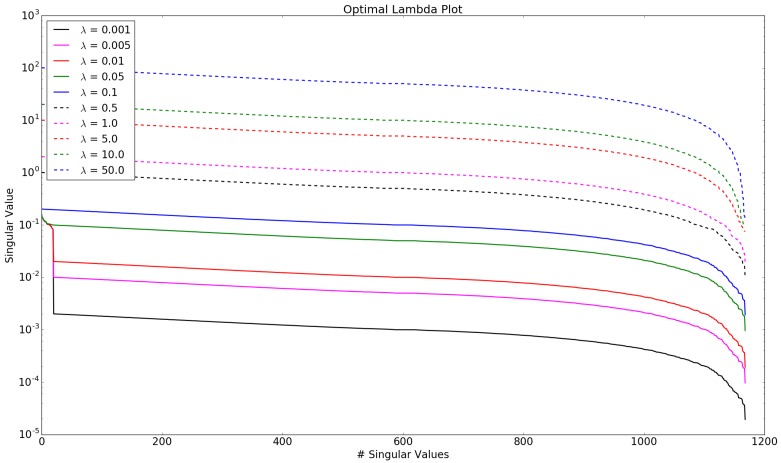
Variation of regularization weight parameter λ.

The introduction and correct weighting of prior information are key factors for the reconstruction quality. The best trade-off between the extinction of artifacts and over-smoothing has to be found to minimize reconstruction error. One approach published for finding a good guess of the λ parameter is to investigate the singular values of the augmented system matrix [17,18]. Figure 5 shows the plot of the singular values dependent on λ. With increasing λ, the singular values of the regularization matrix become more dominant. If the 20 singular values of the system geometry dominate over the regularization singular values, the resulting reconstruction will have stronger artifacts. If the singular values of the regularization matrix dominate, the reconstruction will be over-smoothed.

### 2.6. Measurement Procedure

**Figure 6:**
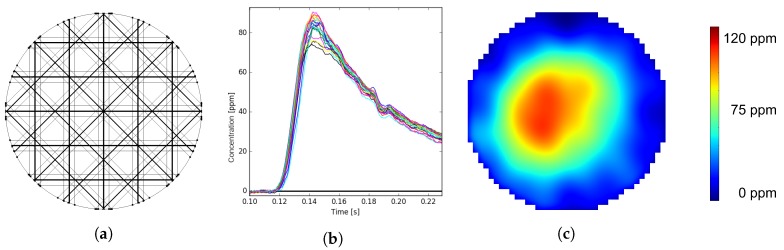
Measurement procedure; (**a**) regular beam arrangement of 20 beams through the cross-section; (**b**) time signal of one injection pulse measuring 20 line concentrations; (**c**) gas distribution after reconstructing the line concentrations at the peak of a injection pulse.

Figure 6 visualizes the measurement procedure beginning with the scanning of the cross-section using the chosen and validated beam arrangement. The acquired line concentrations led to time signals containing 20 line concentrations of ammonia at a high sampling rate. The reconstruction algorithm is then applied on the line concentrations for each time stamp, resulting in an image sequence.

### 2.7. Hot Gas Test Bench

**Figure 7:**
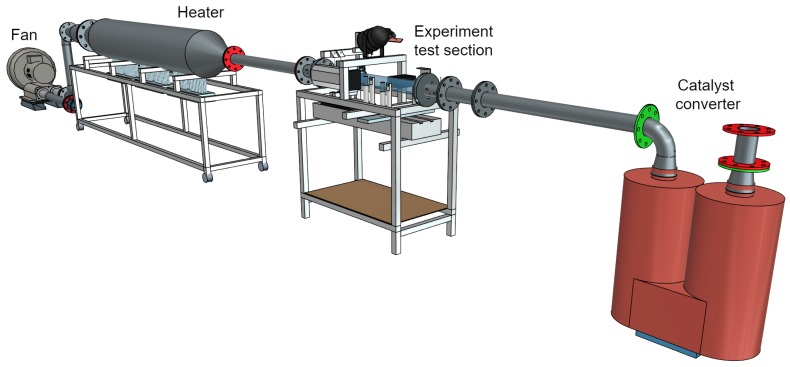
Hot gas test bench enabling various SCR investigations such as depositions, injector mapping, thermology, as well as ammonia concentration distribution measurements using different pipe geometries.

The measurement system is primarily designed for usage on a hot gas test bench operating with hot air instead of exhaust gas. The hot gas test bench (Figure 7) consists of a fan, a heater, the test section containing the UWS injection, as well as various measurement equipment and a catalyst converter for neutralizing the ammonia. To best simulate exhaust gas conditions, the maximum ratings of the test bench allow up to 500 degrees Celsius and a 50-m/s flow speed. The setup enables fundamental investigations on an SCR system such as studying depositions due to unfavorable operating points in terms of temperature or the injection amount of the UWS. Further, detailed thermal analysis of the wall effects due to the impact of fluid injection are investigated. Various pipe geometries are investigated to analyze the effects on the gas distribution and urea conversion rate using the tomographic ammonia detector as the essential measurement system. The high quality and repeatability of the results deliver valuable data for the creation of simulation models and their metrological validation.

## 3. Results

### 3.1. Tomographical Optical Ammonia Detector Prototype

**Figure 8:**
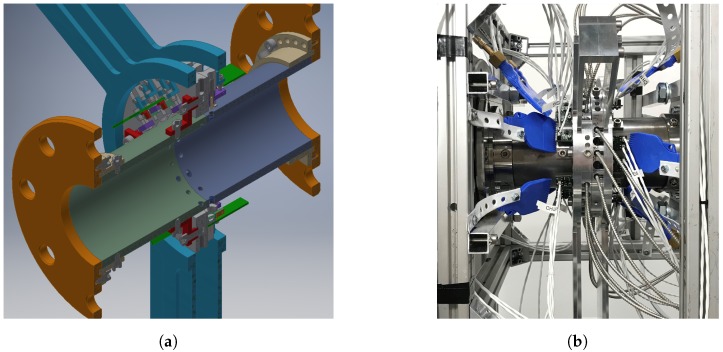
Measurement system prototype; (**a**) CAD sectional model showing the structure of the inner pipe and the detection ring; (**b**) prototype installed on the hot gas test bench measuring the gas distribution of a urea injection experiment.

The first prototype combines all acquired concepts in order to obtain the best measurement results. Figure 8a presents the sectional CAD model showing the exhaust pipe and the holes for optical access. As the pipe has to be gas tight, the holes contain fused silica realized as convex lenses, which already combine the functionality of optical windows with the beam shaping in terms of collimation and converging. The beam arrangement is realized in two measurement planes containing 10 beams each, rotated 45 degrees to each other. The two measurement planes are closely located to each other. Since the gas plume is continuous in flow direction, the systematic error due to separation of the measurement plane stays below one percent, validated with simulation data. A sample rate of 2 kHz results in a cross-section image for approximately every two centimeters of the air stream. A mounting ring containing the detectors and holders for the optical fibers is attached to the pipe with minimum thermal conduction. Since the gas temperature goes up to 400 degrees Celsius, the filters and detectors have to be protected in order to stay within the maximum operating temperature of 50 degrees Celsius. Flat jet nozzles have been added for additional cooling of all sensitive components. The whole prototype is designed to be easily exchanged with a blank pipe replacing the device if not needed. Figure 8b shows the measurement system installed on the hot gas test bench.

### 3.2. Experiment 1: Injection on a Flat Plate

**Figure 9:**
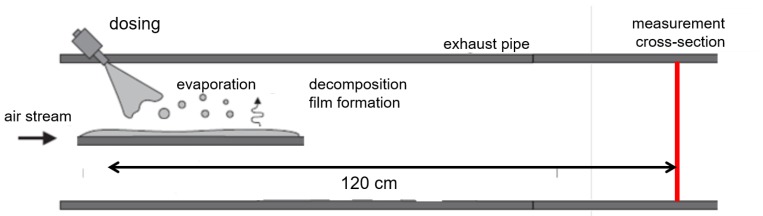
Experiment 1: UWS injection on a hot flat plate within the exhaust pipe.

This experiment is a very suitable setup for validating simulation models since its simple geometry is relatively easy to model. A flat rectangular plate is mounted in the center of the pipe, instrumented with temperature sensors (Figure 9). The SCR injector is mounted to spray directly on the plate. Prior to the injection experiment, the plate has approximately air temperature. At each injection pulse, parts of the UWS evaporate in the air stream and decompose to ammonia through thermolysis and hydrolysis. The main part of the injection hit the flat plate, forming a wall film, which evaporates and decomposes to ammonia. Parts of the injection recoil from the plate and hit the pipe wall, forming a fluid film there as well.

**Figure 10:**
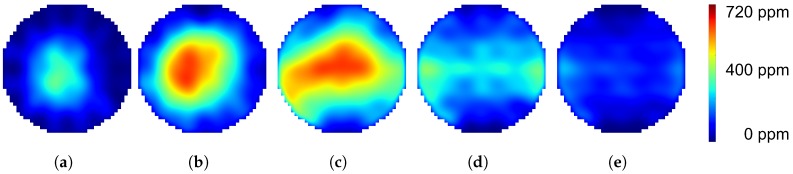
The images (**a**–**e**) show the reconstruction results of measurements from the hot gas test bench. The image sequence visualizes the ammonia concentration distribution of one urea injection pulse on a flat plate in the hot air stream at a distance of 120 cm downstream of the injection spot.

120 centimeters downstream of the injection spot, the cross-section is scanned with the bespoken regular beam arrangement (Figure 6a) leading to 20 line concentration time signals (Figure 6b). After applying the reconstruction algorithm to the time signals, the image sequence in Figure 10 is obtained. This result demonstrates the performance of the measurement system to visualize the dynamic processes during one urea injection. Details like spray formation and conversion in the air stream (Figure 10a,b), as well as evaporation from the flat plate (Figure 10c) and the pipe wall film (Figure 10d) can be clearly identified. Due to the low number of projections through the cross-section, the images contain reconstruction artifacts. These artifacts appear as higher concentrations along the optical paths as mismatches of the reconstruction algorithm clearly visible in Figure 10a. Image smoothing reduces the visible artifacts, but on the other hand, smears and broadens the real concentration distribution. The introduction of more advanced reconstruction methods like the Bayesian approach of modeling may improve this behavior and will be part of future work.

### 3.3. Experiment 2: Injection into an S-Shaped Pipe

**Figure 11:**
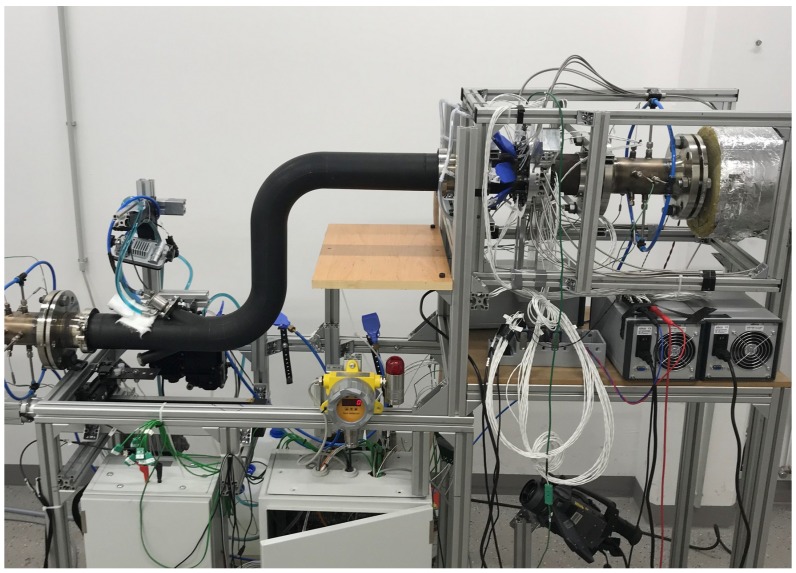
Experiment 2: UWS Injection into an S-shaped pipe.

The S-shaped pipe containing the injection unit represents the geometry of a realistic heavy duty exhaust system (Figure 11). Using a close to reality geometry enables fundamental investigations on wall effects, depositions, thermology, and ammonia distribution. The UWS is injected into the pipe targeting the opposite pipe wall. Thermal analysis illustrates the cooling of the pipe wall and hence the formation of a wall film. The conversion to ammonia takes place within the spray, as well as evaporation from the wall film.

**Figure 12:**
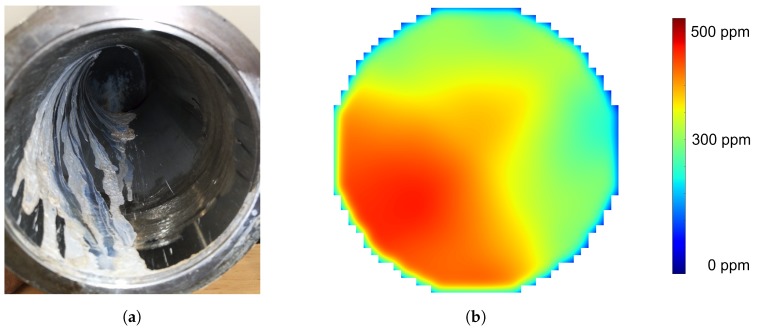
In (**a**) the flow trend of the hot air stream with excessive injection of UWS can be seen. (**b**) shows the correlating ammonia distribution image reconstruction for the same stream conditions, but with less amount of UWS injection. The viewpoint of both images is against the flow direction.

As mentioned above, operating the SCR system in unfavorable operating conditions may lead to depositions inside the exhaust system. Within this experimental setup, the intentionally-generated depositions in Figure 12a visualize the air stream just in front of the measurement cross-section, obtaining the concentration distribution. One can see a helical air stream within the pipe, emphasizing the bottom left pipe region just in front of the measurement cross-section. Figure 12b illustrates the ammonia distribution of an injection pulse at the moment of peak concentration. Temperature and air speed conditions were identical between Figure 12a,b, but with less injection amount. The obtained gas distribution (Figure 12b) correlates very well in location with the witnessed depositions within the pipe (Figure 12a).

## 4. Discussion

The system under test is well validated in each step of development. The detector itself is calibrated and validated with test gas under laboratory conditions in order to guarantee correct ammonia line concentrations. Analysis of the expected reconstruction error rate is executed using test data, obtaining the performance of the chosen regular beam arrangement. All experimental reconstruction results are feasible with respect to the expected gas concentration distributions. The discussed tomographic reconstruction methods work surprisingly well. The chosen beam arrangement is well validated with test data and a good choice in terms of information gain. Least squares regression and Tikhonov regularization are still very simple algorithms. By implementing more advanced methods, like Bayesian modeling approach, there is still potential for reconstruction quality improvement.

First experimental investigations on the measurement prototype indicated a weak point concerning contamination of the lenses. Hence, measurements containing the ammonia detector are selected with low injection amounts to prevent pollution of the lenses. Future development making the system suitable for the engine test bench will cover this problem. The appliance of dispersive techniques using UV spectrometers will eliminate the direct cross sensitivity to lens contamination since pollution will affect the absorbance in a broad spectral range, not affecting the particular spectral shape of the gases to be identified. This technique allows the separation of several gases absorbing in the same spectral region such as NO, NO_2_, or ozone and quantifying them. However, dispersive measurement and the additional functionality of detecting all relevant exhaust gases leads to a reduction of the sampling frequency to a range between 10 and 100 Hz.

## 5. Conclusions

The potential of in situ methods for ammonia concentration distribution in a gas stream has been clearly demonstrated. The low detection limit of about 1 ppm makes the system suitable for ammonia slip measurements after the catalytic converter. Sampling rates up to 3 kHz in combination with the simultaneous scanning of the cross-section result in high temporal resolution snap shots of the ammonia concentration distribution and enables the investigation of dynamic processes for the first time. Compared to extractive methods, the speedup of the in situ approach in the order of 10^7^ is a major improvement for simulation validation and leads to a massive cost reduction when performing measurement campaigns. Making this technology even more powerful, future development will deal with the modification of the system for usage on the engine test bench dealing with real exhaust gas. Changing the detector principle will be mandatory to resolve cross sensitivities, but will add new detection possibilities since all relevant gas species can be identified and quantified. To summarize, future improvement of the SCR technology will need advanced measurement technologies to gain the full potential of NO_X_ reduction in order to fulfill upcoming emission legislation.

## Figures and Tables

**Table 1:** Properties of the three most sensitive ammonia absorption spectral ranges.

	Near-Infrared (NIR)	Mid-Infrared (MIR)	Deep Ultraviolet (DUV)
**spectral region**	1.6 μm	10 μm	210 nm
**absorption coefficient**	10^−21^ cm^2^	10^−18^ cm^2^	2 × 10^−17^ cm^2^
	- -	o	+++
**cost**	++	- -	+
**availability**	wide variety of	hardly any	moderate price,
	materials at a low	temperature-resistant	spectral limit for
	price	materials	fibers and filters
	++	-	o
**cross sensitivity**	H_2_O	hot CO_2_	NO, NO_2_
	-	+	- -

## Abbreviations

The following abbreviations are used in this manuscript:

NH_3_	ammonia
DUV	deep ultraviolet
UV	ultraviolet
NIR	near-infrared
MIR	mid-infrared
TIA	transimpedance amplifier
SNR	signal-to-noise ratio
SCR	selective catalytic reduction
NO_X_	nitrogen oxides
NO	nitric oxide
NO_2_	nitrogen dioxide
H_2_O	water
CO_2_	carbon dioxide
RDE	real driving emission
NEDC	New European Driving Cycle
UWS	urea water solution (AdBlue^®^)
CFD	computational fluid dynamics
CAD	computer-aided design
